# Graphene-Based Catalytic Surfaces Decorated with Au and Cu Nanoparticles: A Model Approach toward e‑Waste-Derived Catalysts

**DOI:** 10.1021/acsmaterialsau.5c00221

**Published:** 2026-07-20

**Authors:** Vittorio Scardaci, Stefano Boscarino, Luca Pulvirenti, Giusy Dativo, Francesco Ruffino, Guglielmo G. Condorelli, Salvatore Scirè, Roberto Fiorenza, Giuseppe Compagnini

**Affiliations:** † Dipartimento di Scienze Chimiche, 9298 Università degli Studi di Catania, Viale A. Doria 6, 95125 Catania, Italy; ‡ Dipartimento di Fisica e Astronomia “Ettore Majorana”, Università degli Studi di Catania, Via S. Sofia 64, 95123 Catania, Italy; § CNR-IMM, Catania (University) Unit, via S. Sofia 64, 95123 Catania, Italy

**Keywords:** graphene, metal nanoparticles, e-waste, CO_2_ conversion, circular economy, laser treatment

## Abstract

The increasing demand for sustainable technologies and resource efficiency is driving the development of materials that can both mitigate CO_2_ emissions and recover valuable metals from e-waste. In this work, we report a simple and environmentally friendly approach for fabricating catalytic surfaces based on laser-reduced graphene oxide (rGO) decorated with gold or copper nanoparticles (Au or Cu NPs). The process involves laser reduction of graphene oxide, followed by immersion in aqueous Au^3+^ or Cu^2+^ model solutions, simulating metal leachate from an e-waste recovery process. Morphological and chemical analyses (SEM and XPS) confirm the formation of metallic nanoparticles. The resulting metal@rGO surfaces were tested for CO_2_ photothermo-catalytic conversion under simulated solar irradiation, achieving conversion efficiencies above 40%, with CO and CH_4_ as the main products. Both catalytic surfaces displayed comparable activity and good stability over three consecutive cycles. This method integrates CO_2_ conversion with metal recycling, offering a promising route toward circular catalytic materials.

## Graphical abstract

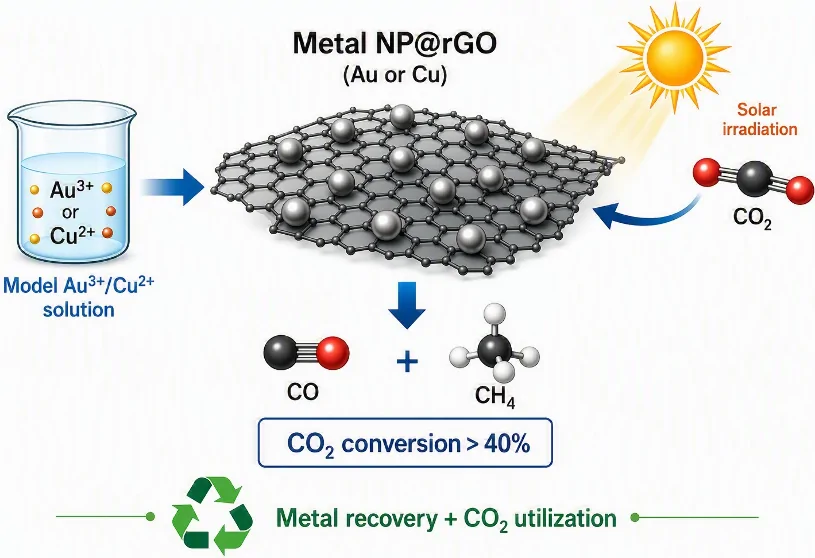

## Introduction

Environment and sustainability issues are becoming more and more relevant nowadays, and resource efficiency is the focus of significant efforts worldwide, with noble metals playing a pivotal role, pushing toward the development of a circular economy.^1,2^ Indeed, waste generated from electrical or electronic equipment, or e-waste, is a rising issue, posing several challenges on developed economies.^3^ On the other hand, efforts to tackle CO_2_ emissions are developing very fast and are a hot topic in the scientific community.^4,5^

Recovering metals from e-waste typically requires a strong acid attack that causes metal oxidation and dissolution in the liquid medium as metal ions. This is then to be followed by separation and reduction.^6,7^ Recently, reduced graphene oxide (rGO) has been proposed as a viable candidate material for the recovery of gold from e-waste in the liquid phase, leveraging the free charges intrinsically available within such graphene-based material.^8^

Beside chemical reduction in liquid, a fast and cheap laser-based reduction of graphene oxide exists, which is mainly carried out on a surface in air, controlled atmosphere or in the liquid phase.^9–12^ The process is based on the local laser-induced thermal heating that causes the release of oxygenated groups from graphene oxide.^9,10^ Such method has the advantage of being faster, potentially cheaper and more environment friendly than chemical reduction methods, due to the absence of reducing chemicals involved. These laser-based methods have been already demonstrated for patterning, sensing, energy storage and surface treatment applications.^13^

Tackling CO_2_ emissions may involve strategies of capture, conversion, or a combination of both.^4,14^ CO_2_ capture methods are mainly designed to separate and store CO_2_ from fuel gases or directly from the atmosphere, while conversion approaches focus on transforming CO_2_ into value-added chemicals and fuels.^15^ Among the latter, catalytic methods are particularly attractive since they enable the activation of this thermodynamically stable molecule under more sustainable conditions. Depending on the energy input, these processes can be classified into photocatalysis, thermocatalysis, and photothermo-catalysis, each relying on different mechanisms to drive the conversion reaction.^16^ Photocatalysis exploits solar photons to generate charge carriers capable of reducing CO_2_, thermocatalysis requires heat to activate the reaction pathways, whereas photothermo-catalysis combines both light and thermal energy, offering synergistic effects that often result in higher efficiency and selectivity.^17–21^ This integration of capture and catalytic conversion within the broader Carbon Capture and Utilization (CCU) framework represents a promising route toward mitigating emissions while simultaneously producing renewable fuels and chemicals.^22^

Photothermo-catalysis has emerged as a promising strategy within the broader framework of Carbon Capture and Utilization (CCU), enabling the sustainable transformation of CO_2_ into value-added fuels and chemicals by synergistically combining solar light and thermal energy.^22,23^ This dual-activation approach enhances charge separation and accelerates surface reactions, addressing key limitations of conventional photocatalysis.^17^ Recent studies^24–28^ have shown that hybrid nanomaterials can significantly boost CO_2_ conversion efficiency under photothermal conditions and these systems pave the way toward efficient solar-to-chemical energy conversion while contributing to a circular carbon economy.

The incorporation of reduced graphene oxide (rGO) and metal nanoparticles in the catalytic system plays a crucial role in enhancing the photothermo-catalytic conversion of CO_2_. The high electrical conductivity of rGO facilitates charge carrier separation and electron transport from the photoactive component to the catalytic sites, effectively suppressing charge recombination^29–32^

Metal nanoparticles contribute both through their intrinsic catalytic activity for CO_2_ activation and through localized surface plasmon resonance (LSPR), which generates hot electrons upon visible light irradiation.^33,34^ These hot electrons can be transferred to the conduction band of the adjacent semiconductor or directly participate in the reduction of CO_2_, promoting the formation of CO and CH_4_.^35–37^ For example, the synergistic combination of rGO with Au or the more economic Cu results in improved charge separation, enhanced light harvesting, and efficient activation of CO_2_, making the hybrid system highly effective for photothermo-catalytic applications.^38,39^

In this letter, we present an integrated approach in which a catalytic surface is obtained from laser-reduced graphene oxide, functionalized by gold or copper nanoparticles (Au or Cu NPs) obtained by exposure to an Au^3+^ or Cu^2+^ model solution, mimicking what can be obtained as a leachate from an e-waste metal recovery process. The surfaces were characterized by electron microscopy and X-ray Photoelectron Spectroscopy (XPS). Finally, such surfaces are employed in the photothermo-catalytic conversion of CO_2_ into methane and CO, showing >40% conversion and reusability up to three times (more than 15 h of simulated solar irradiation at 120 °C) without significant loss of conversion efficiency. While real e-waste leachates are significantly more complex systems, typically containing multiple metal ions, residual acids, and organic species, the use of model solutions allows studying the individual metal deposition process onto GO/rGO systems, as well as their relative catalytic performance, making them suitable platforms for a proof-of-concept study.

## Materials and Methods

Graphene oxide (GO) solution (0.4 wt %) was purchased from Graphenea and used as received. Laser reduction was performed using a Qiilu DK-BL machine, mounting a continuous 405 nm laser with 1.5W nominal power. The combined motion of the laser and the substrate makes a raster movement of the laser onto the surface, as designed by software. The reduction process was typically performed at ∼6 mm/s scan speed.

To obtain metal NPs on the rGO surface, 250 μL of the GO solution is drop-casted onto Cu tape (1 cm × 1 cm), applied onto a glass slide, and dried at room temperature overnight under fume-hood ventilation. Such substrate is exposed to the laser scribing process, as described in a previous work.^11^ After reduction, the substrate is immersed in 10 mL of a 2.5 × 10^–6^ M AuCl_3_ or a 10^–4^ M CuSO_4_ aqueous solution for 1h, before being rinsed and dried. See Figure S1 for an image of a typical sample. Samples of metal NPs on GO were also prepared, skipping the laser scribing process.

SEM images were acquired by a field-emission Gemini 152 Carl Zeiss Supra 25 microscope. The analyses were carried out by using the in-lens detector with an aperture size of 30 μm, a working distance of 3 mm, and an acceleration voltage of 3 kV. Statistical analysis of particle size distribution was carried out using the ImageJ software.

XPS measurements were carried out using a PHI 5000 Versa Probe Instrument with a monochromatic Al Kα X-ray source and at a photoelectron takeoff angle of 45 (relative to the sample Surface).

Photothermo-catalytic tests were performed as reported in ref 22. Specifically, a cylindrical batch Pyrex reactor was used, in which the film catalyst sample was placed. The system was irradiated for 5 h using a solar lamp (Osram Ultra Vitalux, 300 W) providing an irradiance of 10.7 mW/cm^2^. The reactor was put into an insulated special solar box, where the temperature reached 120 °C. This temperature was continuously monitored and controlled using an infrared thermal camera. A gaseous mixture of CO_2_ and H_2_O vapor was introduced into the reactor to saturate the catalyst surface with the reactant molecules. The water vapor was generated using a water bubbler maintained at 80 °C and mixed with high-purity CO_2_ (99.999%). The CO_2_/H_2_O flow rate was regulated by mass flow controllers to achieve a molar ratio of 15:1, in order to promote CO_2_ reduction over the competing water splitting reaction.

The reaction products were analyzed using gas chromatography. CO and CH_4_ were detected as the only reaction products obtained in the used experimental conditions, and they were quantified using a gas chromatograph (GC, Agilent 8860) equipped with a CARBOXEN-1000 column and a thermal conductivity detector (TCD), opportunely calibrated for the detection of carbon dioxide, methane and carbon monoxide.

The CO_2_ conversion values were determined using the following equation.

CO_2_ conversion (%):

$$\left(\frac{{\mathrm{Area}}_{\mathrm{peak CO}2\;\mathrm{in}}{-\mathrm{Area}}_{\mathrm{peak CO}2\;\mathrm{out}}\times \left(\frac{{\mathrm{Area}}_{\mathrm{peak standard in}}}{{\mathrm{Area}}_{\mathrm{peak standard out}}}\right)}{{\mathrm{Area}}_{\mathrm{peak CO}2\;\mathrm{in}}}\right)\times 100$$

Furthermore, also the mass balance method was applied, considering the following equation.

CO_2_ conversion (%):

$$\left(\frac{{\mathrm{Area}}_{\mathrm{peak CO}2\;\mathrm{out}}}{{\mathrm{Area}}_{\mathrm{peak products out}}+{\mathrm{Area}}_{\mathrm{peak CO}2\;\mathrm{out}}}\right)\times 100$$

The two methods were in accordance with each other (±5%, reproducibility 95%).

The molar selectivity toward CO and CH_4_ products was evaluated with eqs 1 and 2:

1 $${\mathrm{CH}}_{4}\;\mathrm{selectivity \%:}\;\left(\frac{N_{CH4}}{\left(N_{CH4}+N_{CO}\right)}\right)\times 100$$

2 $$\mathrm{CO selectivity \%:}\;\left(\frac{N_{CO}}{\left(N_{CH4}+N_{CO}\right)}\right)\times 100$$

where N_CH4_ and N_CO_ are the production rates of CH_4_ and CO in μmol/g_catalyst_ h_irradiation_.

Selectivity was calculated on a molar carbon basis, as appropriate for photocatalytic systems where no external current is applied. Faradaic efficiencies are not applicable in the absence of an electrochemical circuit.

## Results and Discussion

Figure 1 shows representative Scanning Electron Microscopy (SEM) images of the samples analyzed in this work. Size particles, determined by carrying out statistical analysis on the SEM images, are summarized in Table 1. We can observe that exposure of rGO to both model solutions yields ∼2× larger particles than GO exposure to the same solutions. Au@rGO films also show much larger particles, in the hundreds of nm range. Also, a larger number of particles is formed on rGO. This suggests a higher reduction efficiency of rGO.

**Table 1:** Size Distribution of Au and Cu NPs on GO and rGO

Sample	Size (nm)	StDev	N
Au@GO	34	±9	74
Au@rGO	70	±18	70
Cu@GO	34	±11	70
Cu@rGO	64	±20	93

**Figure 1:**
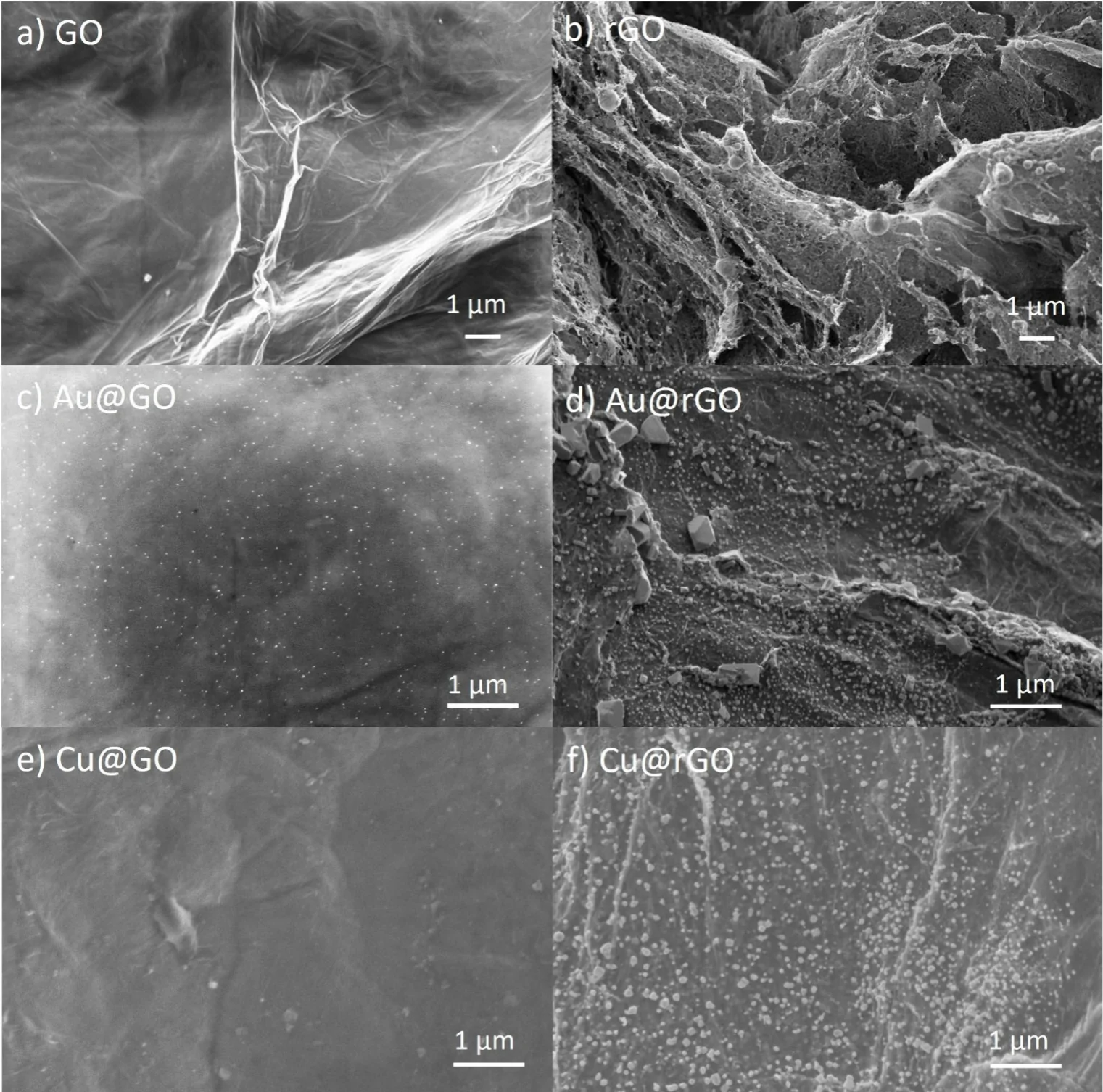
Representative SEM images of samples analyzed in this work.

The chemical composition of our GO/NP surfaces was assessed by XPS analysis. As expected, The C 1s spectra of unreduced and reduced GO show that the C–O and C=O components are significantly reduced in intensity for reduced GO (Figure S2), while the C=C component remains unaltered. The quantitative atomic percentages obtained from XPS survey spectra are summarized in Table 2.

**Table 2:** Atomic Composition (XPS) of GO–AuCu and rGO–AuCu Samples

Sample	C (%)	O (%)	Cu (%)	Au (%)
GO–AuCu	65.9	33.5	0.6	0.1
rGO–AuCu	84.0	14.6	1.1	0.3

In Au@GO/rGO samples, the Au 4f spectra of the two samples show an Au 4f_7/2_–Au 4f_5/2_ doublet, where the main component at approximately 84.0 eV is attributable to metallic Au^0^ (Figure 2a,b). The corresponding Au 4f5/2 component is observed at approximately 87.7 eV, consistent with the characteristic spin–orbit splitting of metallic gold reported in the literature.^40,41^

**Figure 2:**
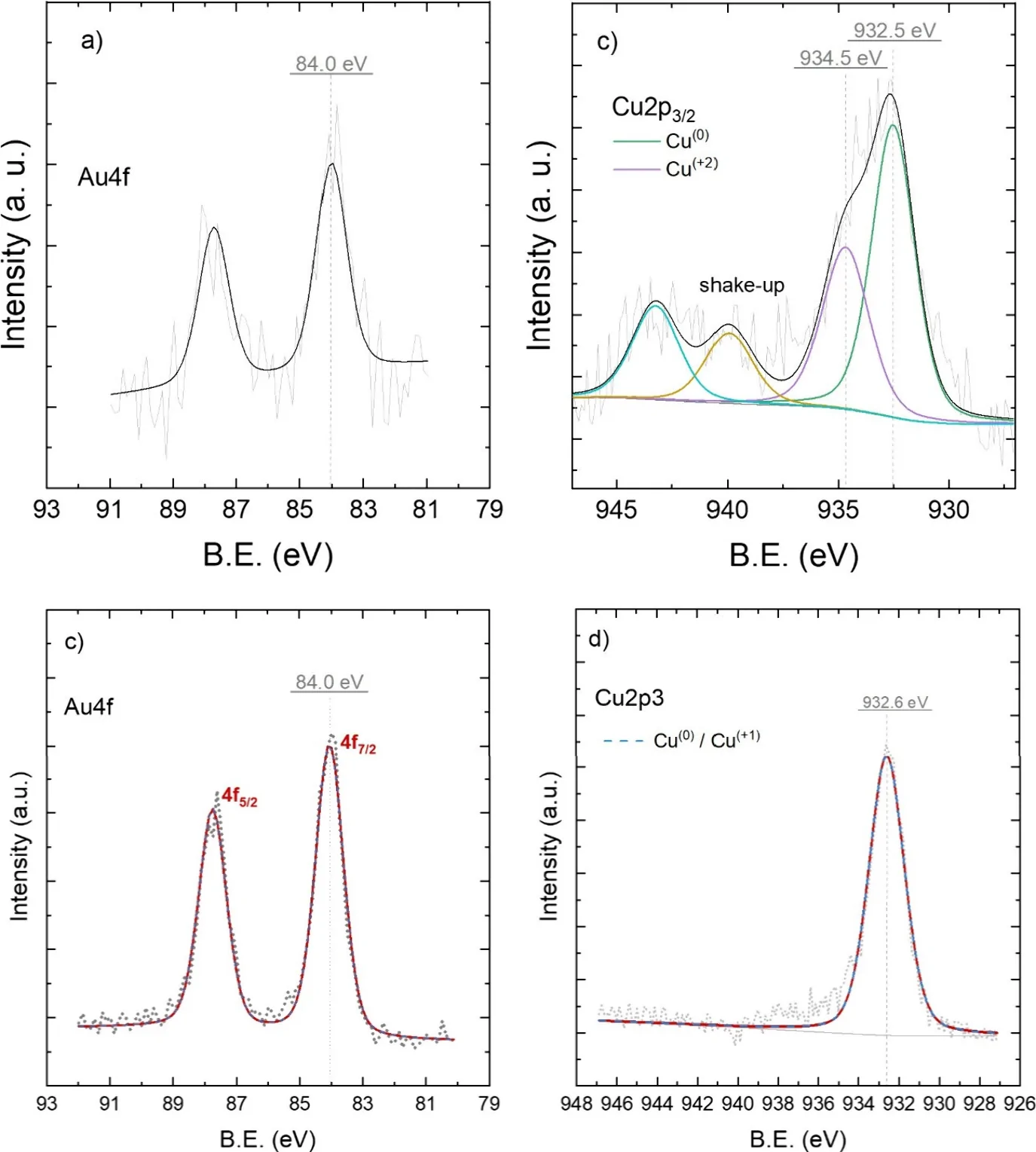
XPS spectra of (a) Au@GO; (b) Au@rGO; (c) Cu@GO; (d) Cu@rGO.

In the Cu@GO sample, the Cu 2p_3/2_ spectrum shows two distinct components at approximately 932.5 and 934.5 eV (Figure 2c). The 932.5 eV component is attributable to Cu^0^ or Cu­(I), while the 934.5 eV component and the characteristic shakeup peaks at 940–945 eV indicate the presence of Cu^2+^, likely in the form of surface CuO. The CuLMM Auger spectra (Figure S4a) display two peaks at 568.9 and 571.9 eV, consistent with the coexistence of copper species in different oxidation states. The data confirm that copper is present in both metallic and surface-oxidized forms. In the Cu@rGO sample, the Cu 2p_3/2_ spectrum consists of a single peak centered at approximately 932.6 eV (Figure 2d), without evidence of shakeup, indicating the absence of significant Cu^2+^ and suggesting that copper is mainly in reduced states (Cu^0^ and/or Cu^1+^). The Cu LMM spectra (Figure S4b) show two peaks at 568.0 and 571.0 eV (BE), slightly shifted compared to the Cu@GO sample, consistent with a more reduced state of copper. Together, the Cu 2p and Cu LMM data indicate that the reduction of GO favors the presence of Cu^0^ and Cu­(I), with less surface oxidation than in the Cu@GO sample.

The quantitative atomic composition reported in Table 1 further supports these findings. In particular, the increase in the C/O ratio from GO to rGO films confirms the effective reduction of graphene oxide, while the detection of Au and Cu in both samples is consistent with the successful deposition of metal nanoparticles.

Figure 3 compares the photocatalytic CO_2_ reduction performance of GO- and rGO-based catalysts decorated with Au and Cu NPs. In all cases, nanoparticle incorporation enhances CO and CH_4_ production as well as CO_2_ conversion compared to the bare carbonaceous supports. Among the tested systems, rGO consistently shows superior activity over GO, confirming that its higher conductivity and lower oxygen content favor charge transport and photocatalytic efficiency.^30,42^ When comparing Au and Cu NPs, both metals yield very similar improvements, with only a slight improvement observed for Au. In particular, Au@rGO reaches the highest CO_2_ conversion (43.8%), closely followed by Cu@rGO (42.8%).

**Figure 3:**
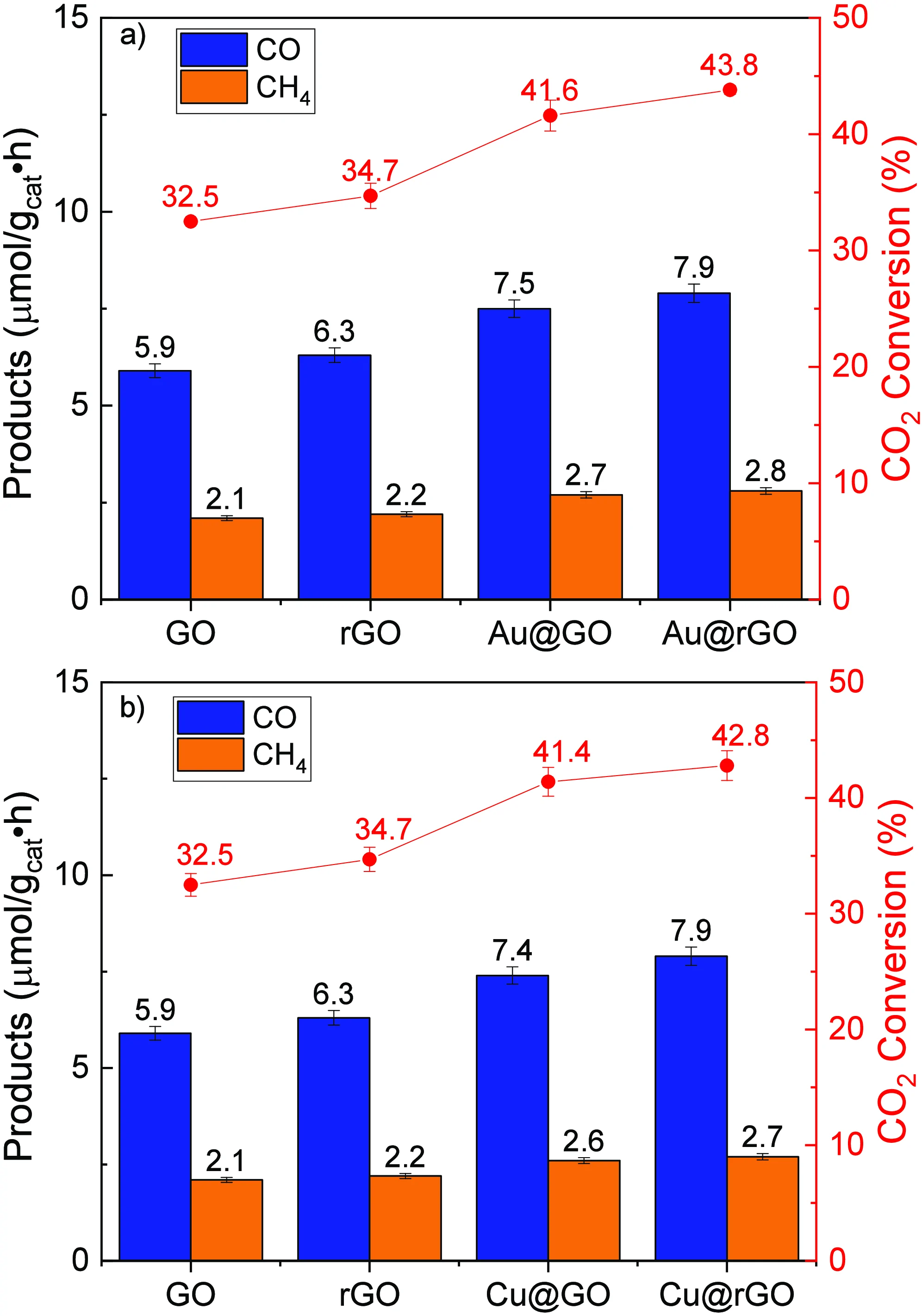
CO_2_ conversion rates, in μmol of formed CO/CH_4_ per g of the used catalyst per h of irradiation, under solar photothermo-catalytic conditions for GO/rGO surfaces, with/without Au NPs (a) and Cu NPs (b).

Indeed, the addition of Au and Cu to rGO in photothermocatalytic CO_2_ conversion offers distinct and synergistic benefits due to their unique physicochemical properties. Copper oxides (CuO, Cu_2_O) are well-known for their catalytic activity in CO_2_ reduction, especially facilitating the formation of hydrocarbons such as methane and carbon monoxide via enhanced adsorption and activation of CO_2_ molecules.^43,44^ On the other hand, Au NPs benefit from plasmonic effects that promote light absorption and charge separation, generating hot electrons and holes that can be transferred to adjacent catalytic sites, thus amplifying photoreactivity.^45^

In our experimental conditions, only CO and CH_4_ were obtained after 5 h of solar photothermo-catalytic tests. Considering the molar selectivity (see the Materials and Methods section), the selectivity did not change among the examined samples with a 74% of selectivity toward CO and 26% into CH_4_, pointing to a predominant two-electron reduction pathway, as also confirmed in the literature under similar experimental conditions.^22,46^

However, the overall CO_2_ conversion remains below 50%, mainly due to the use of catalysts in film form, which inherently limits the exposed surface area and gas–solid interaction compared to powder systems.^47,48^ Note that the bare Cu tape did not show any photothermo-catalytic activity. Conversely, considering that the reaction was conducted at 120 °C and under simulated solar irradiation, the CO_2_ conversion values obtained with the rGO-based samples are promising, taking into account that for the bare thermocatalytic approach a temperature higher than 200 °C is required to activate the CO_2_ molecules.^49,50^ Indeed, it is important to highlight that the CO_2_ conversion in the bare photocatalytic approach (room T, only solar light) was negligible as well as no activity was detected in the dark experiments even heating up to 120 °C (reached temperature in the photothermo-catalytic approach). While it may seem surprising that both Au and Cu NPs yield very similar conversion efficiency when coupled to GO and rGO, it is important to note that the catalytic process is driven by the GO/rGO component, while Au and Cu NPs, through their plasmonic effect, enhance the light absorption in the visible spectrum, increasing the GO/rGO catalytic efficiency to a similar extent.

The product distribution reveals a clear preference toward CO formation over CH_4_. On a molar carbon basis, CO accounts for 73.8% of the carbon-containing products, while CH_4_ represents 26.2%. This selectivity trend suggests that the two-electron reduction pathway of CO_2_ to CO is kinetically favored over the more demanding eight-electron pathway leading to CH_4_. The formation of CO requires fewer proton-coupled electron transfer (PCET) steps and involves lower kinetic barriers compared to the multielectron, multiproton reduction to CH_4_. Consequently, under the present photocatalytic conditions, charge carrier availability and lifetime likely favor partial reduction rather than deep hydrogenation of CO_2_. Moreover, CH_4_ formation typically requires efficient surface hydrogenation steps and sustained electron accumulation, which may be limited by charge recombination and/or insufficient surface hydrogen coverage. Therefore, the predominance of CO indicates that the catalyst promotes selective C–O bond cleavage without extensive C–H bond formation, pointing to a mechanistic regime dominated by early stage reduction intermediates (e.g., *COOH or *CO species). These findings are consistent with a photocatalytic system in which charge separation efficiency and surface reaction kinetics govern the competition between two-electron and multielectron pathways.

To assess the stability of our platforms, we repeated photothermocatalytic tests and observed that the samples maintained their activity after 3 consecutive runs of at 120 °C under simulated solar irradiation (Figures S5 and S6). After that, the films lost adhesion to the substrate and curled up. For this reason, we could only repeat SEM and XPS measurements after the first run. We show that film morphology is unchanged after the photothermocatalytic test (Figure S7). XPS shows that films are also chemically stable, as spectra are mainly unchanged. In particular, Au 4f spectra remain unchanged, suggesting that Au maintains its metallic form with no evidence of oxidation (Figure S8). The Cu 2p_3_/_2_ spectrum shows a main component centered at about 933.8 eV, accompanied by the appearance of shakeup satellites characteristic of Cu^2+^ species (Figure S9). This result highlights a partial surface oxidation of copper. However, this is likely coming from exposure to the model aqueous solution used to deposit the nanoparticles, before the photothermo-catalytic test itself.

While future efforts will have to be devoted to the improvement of substrate adhesion, in order to extend the reusability of our platforms, this configuration is particularly promising for technological applications, such as device integration and structured reactors, making these results highly promising for practical CO_2_ utilization strategies.

## Conclusions

In summary, we developed a method to reduce Au^3+^ and Cu^2+^ to AuNP and CuNP using a laser reduced GO surface. This method is compatible with Au and Cu recycling from e-waste. Such surface is further utilized as a catalyst to convert CO_2_ into methane and CO, a process compatible with industrial processes where such chemicals are used. While at this stage the reusability is only feasible up to three times, we believe this is mainly due to adhesion of the NP@rGO material to the substrate rather than the material itself, hence further development efforts should be devoted to testing a broader range of substrates and improving material adhesion to them. It is important to note that real e-waste derived systems present a higher level of chemical complexity than the model solutions used in this work, which may influence both nanoparticle formation and catalytic performance. Future work will thus focus on applying the present approach to real leachates, including strategies like ion separation and codeposition. In a broader perspective of circularity, our process is able to contribute to CO_2_ emission reduction while at the same time lowering the impact of e-waste by using recycled gold and copper. This concept may be further expanded to the utilization of graphene obtained from natural sources.

## Supplementary Material



## Notes

The supporting material includes: photo image of a typical sample (S1), XPS spectra of GO and rGO (S2), XPS survey of Au@RGO and Cu@RGO samples (S3), XPS Auger spectra for Cu@GO and Cu@rGO (S4), CO_2_ conversion rate and CO/CH_4_ yield for Au@RGO samples across three consecutive runs (S5), CO_2_ conversion rate and CO/CH_4_ yield for Cu@RGO samples across three consecutive runs (S6), SEM images for Au@RGO and Cu@RGO before and after the catalytic process (S7), high-resolution Au 4f XPS spectrum of the Au@rGO catalyst acquired after the photothermocatalytic CO_2_ conversion tests (S8), High-resolution Cu 2p XPS spectrum of the Cu@rGO catalyst acquired after the photothermocatalytic CO_2_ conversion tests (S9) The Supporting Information is available free of charge at https://pubs.acs.org/doi/10.1021/acsmaterialsau.5c00221.
