# Supplementary material for: Graphene-Based Catalytic Surfaces Decorated with Au and Cu Nanoparticles: A Model Approach toward e‑Waste-Derived Catalysts
Source: ACS Mater Au. 2026 Jul 20;6(5):1019–25. doi: 10.1021/acsmaterialsau.5c00221 (PMC13564173; doi:10.1021/acsmaterialsau.5c00221)

Graphene-Based Catalytic Surfaces Decorated with Au and Cu Nanoparticles: A Model Approach Toward e-Waste–Derived Catalysts

Vittorio Scardaci[1,*], Stefano Boscarino[2], Luca Pulvirenti[1], Giusy Dativo[1], Francesco Ruffino[2,3], Guglielmo G. Condorelli[1], Salvatore Scirè[1], Roberto Fiorenza[1], Giuseppe Compagnini[1]

[1]Dipartimento di Scienze Chimiche, Università degli Studi di Catania, Viale A. Doria 6, 95125 Catania, Italy

[2]Dipartimento di Fisica e Astronomia "Ettore Majorana", Università degli Studi di Catania, Via S. Sofia 64, 95123 Catania, Italy

[3]CNR-IMM, Catania (University) Unit, via S. Sofia 64, 95123 Catania, Italy

*Email: vittorio.scardaci@unict.it

SUPPLEMENTARY MATERIAL

Figure S1. Photo image of a typical sample

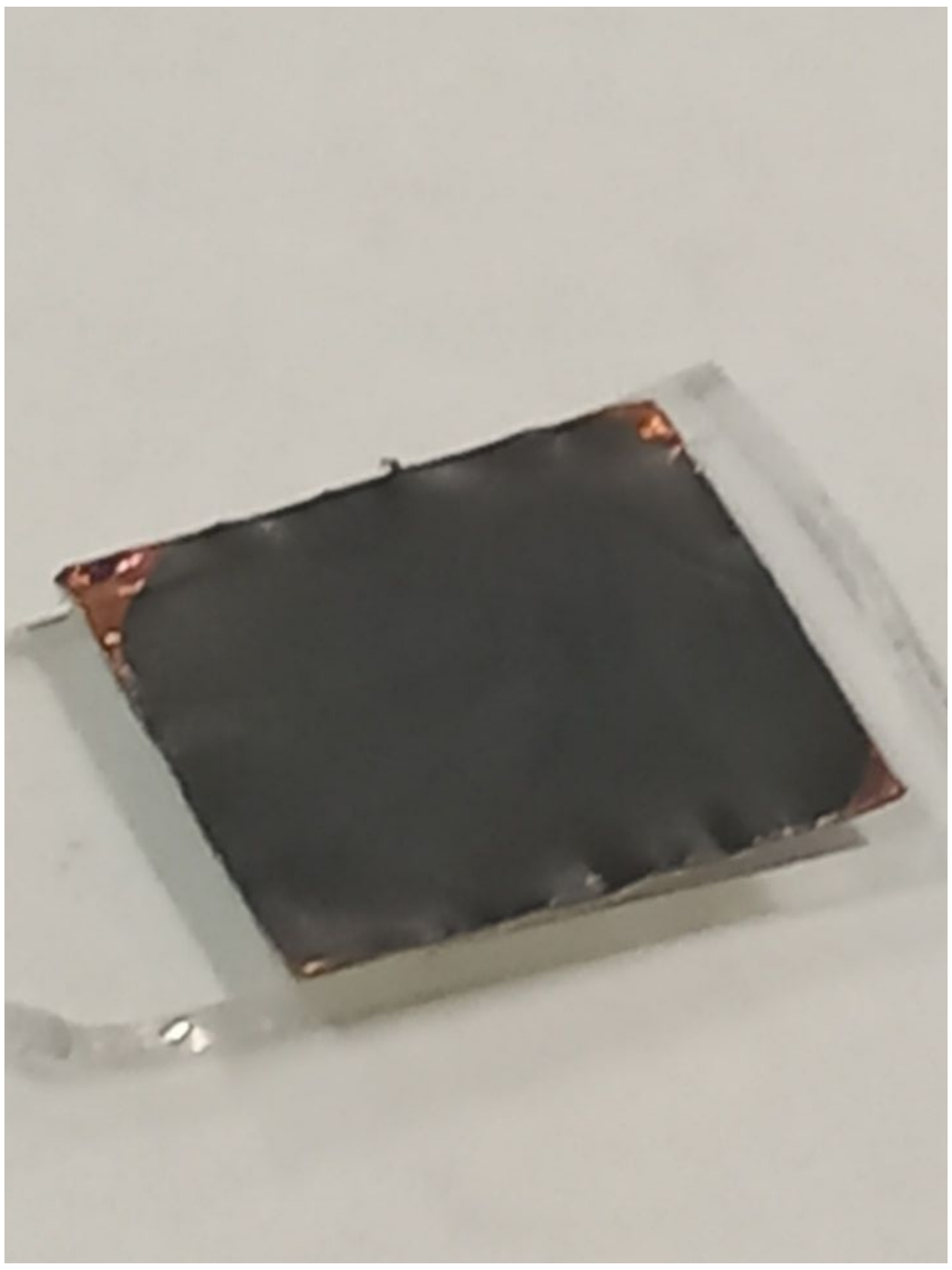

Figure S2. XPS spectra of a) graphene oxide (GO) and b) reduced graphene oxide (rGO)

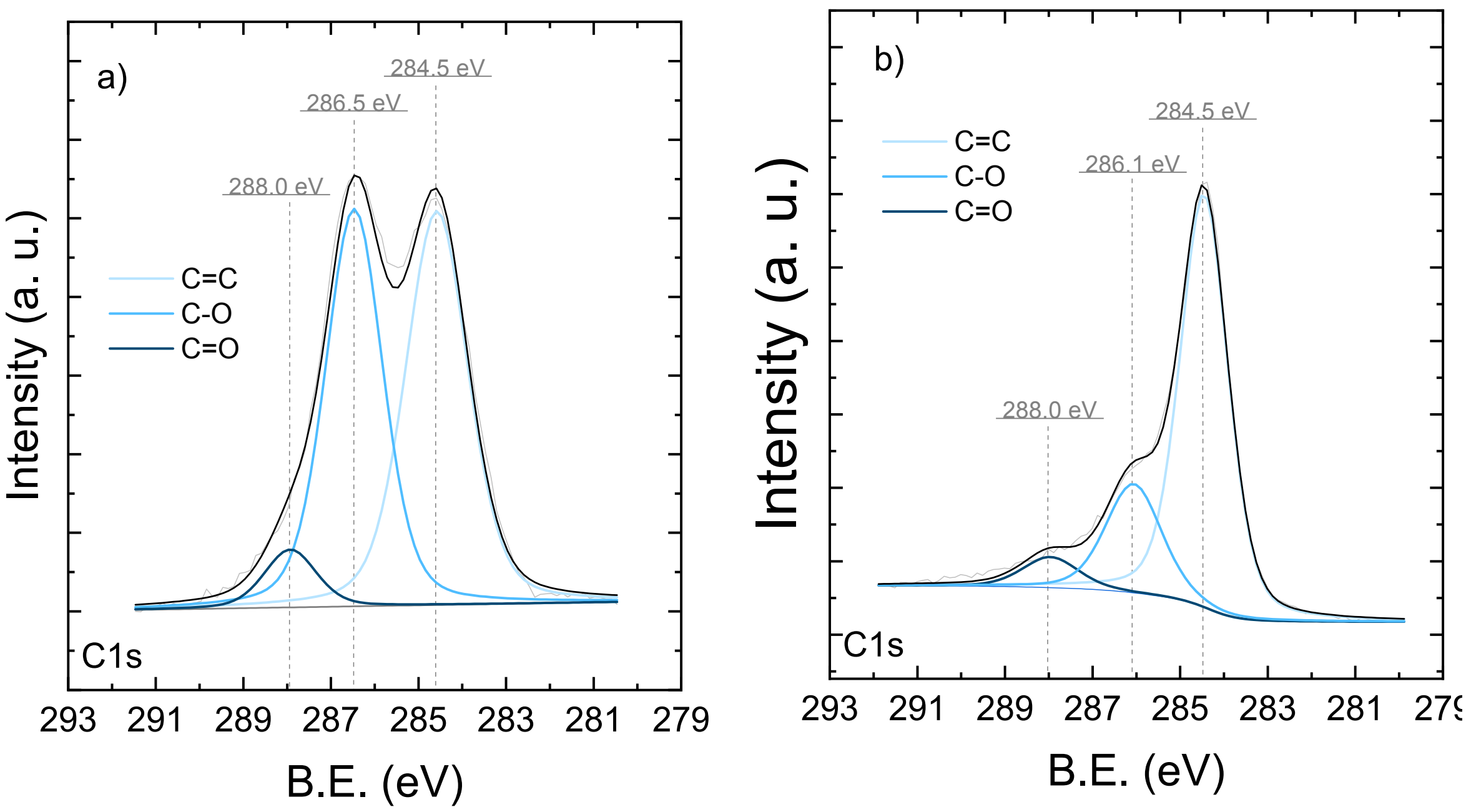

Figure S3: XPS survey of Au@RGO and Cu@RGO samples

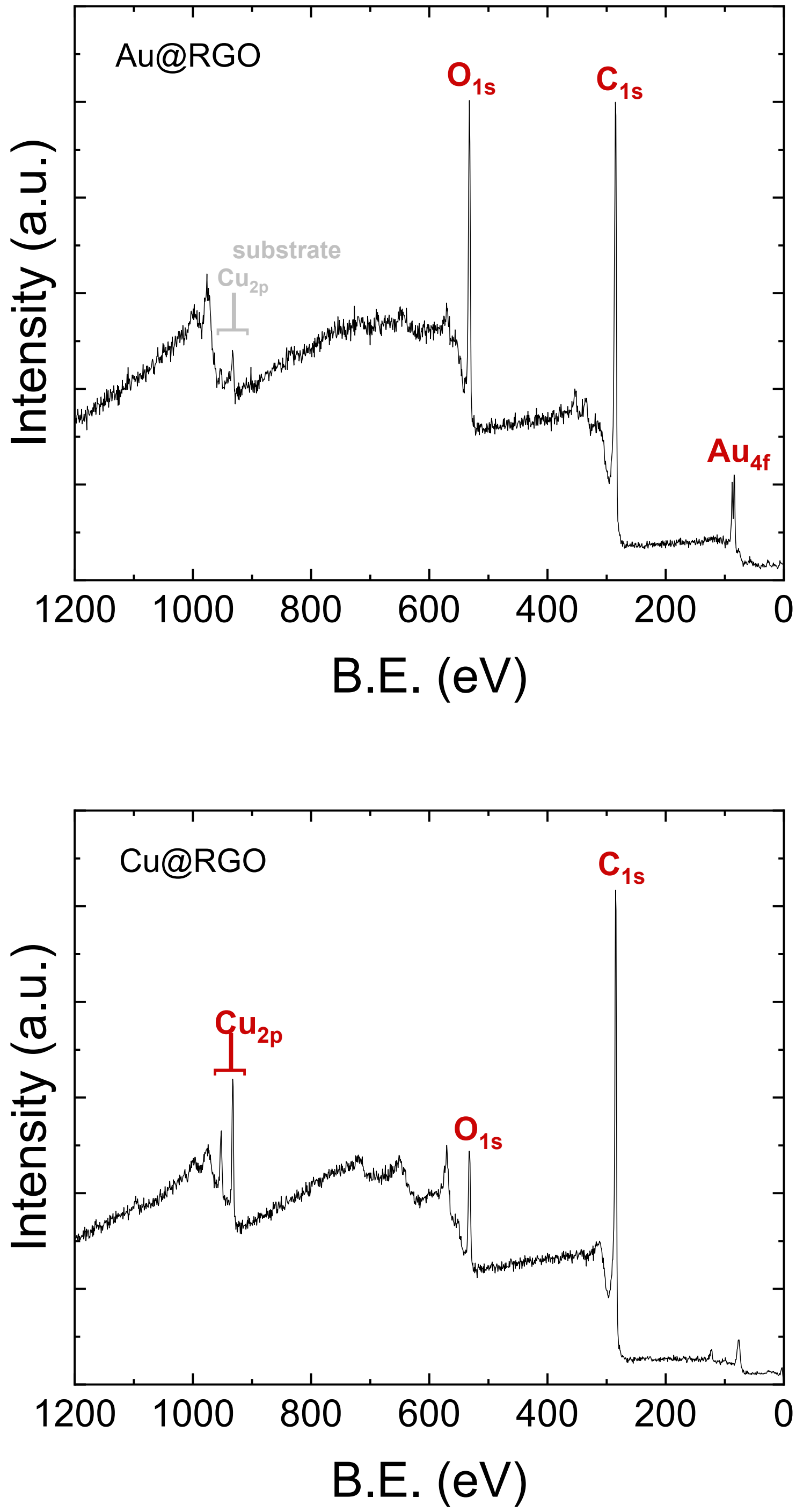

Figure S4: XPS Auger spectra for Cu@GO and Cu@rGO

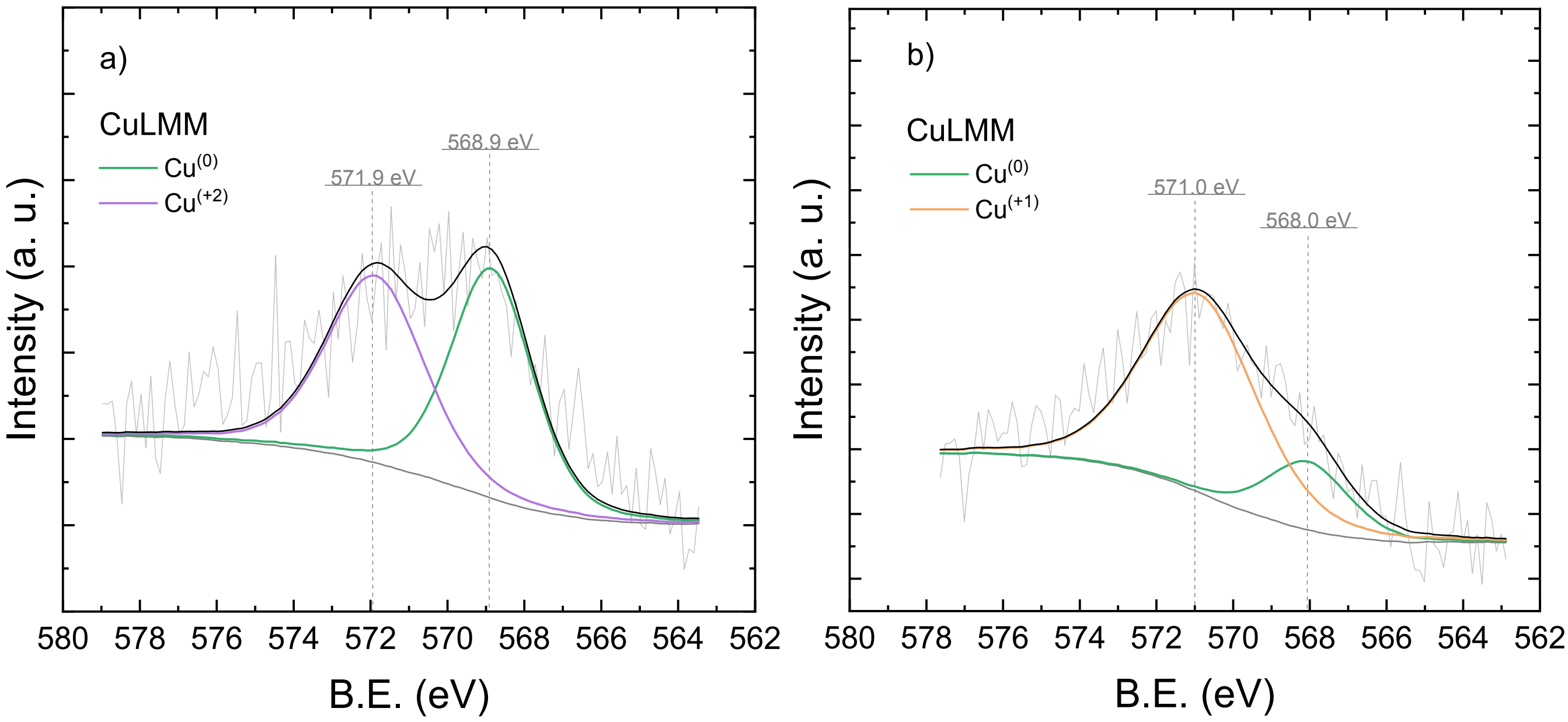

Figure S5: CO_2 conversion rate and CO/CH_4 yield for Au@RGO samples across three consecutive runs (15 h of simulated solar irradiation at 120°C)

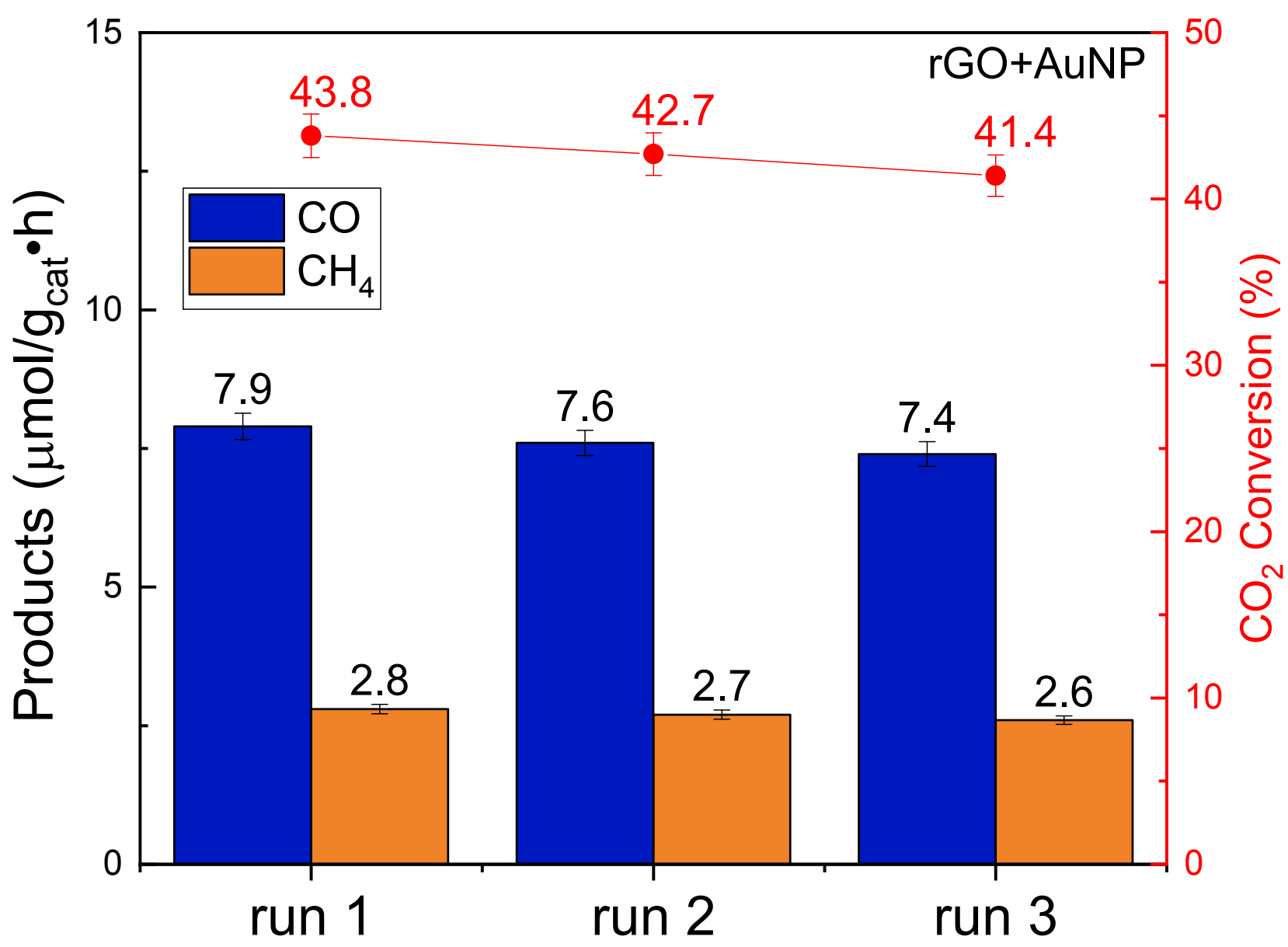

Figure S6: CO_2 conversion rate and CO/CH_4 yield for Cu@RGO samples across three consecutive runs (15 h of simulated solar irradiation at 120°C)

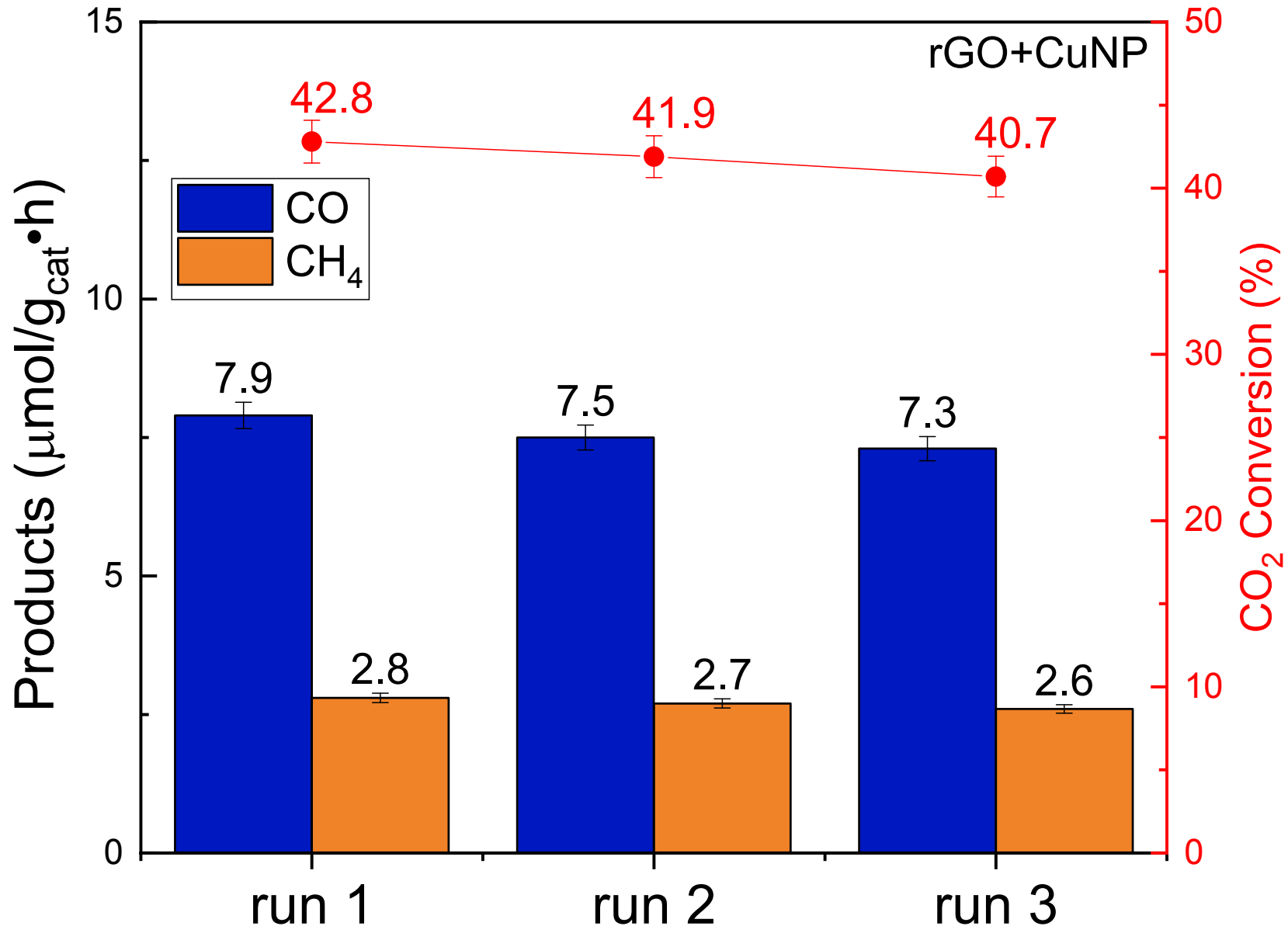

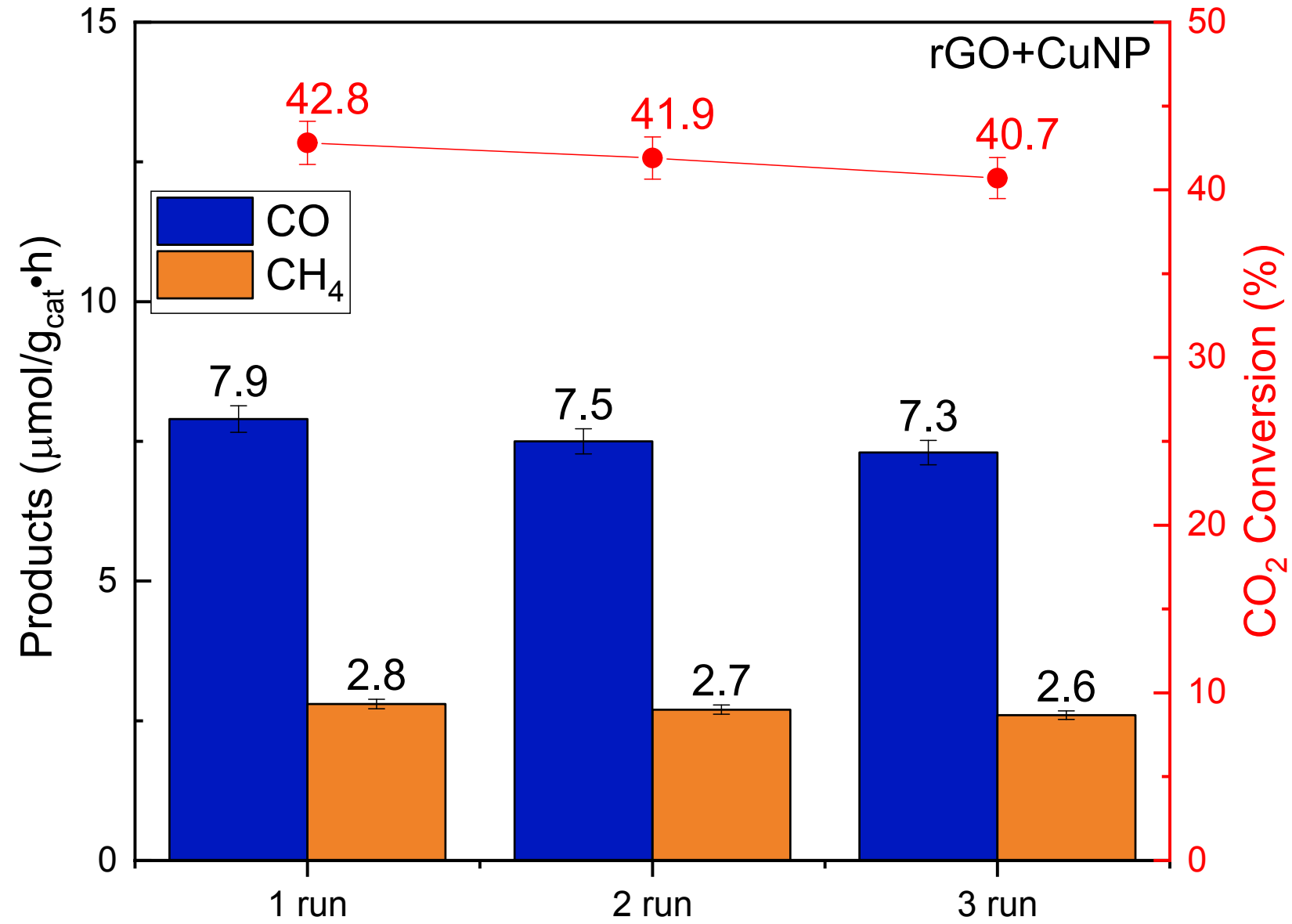

Fig. S7. SEM images for a) Au@RGO before, b) Au@RGO after, c) Cu@RGO before, d) Cu@RGO after the catalytic process

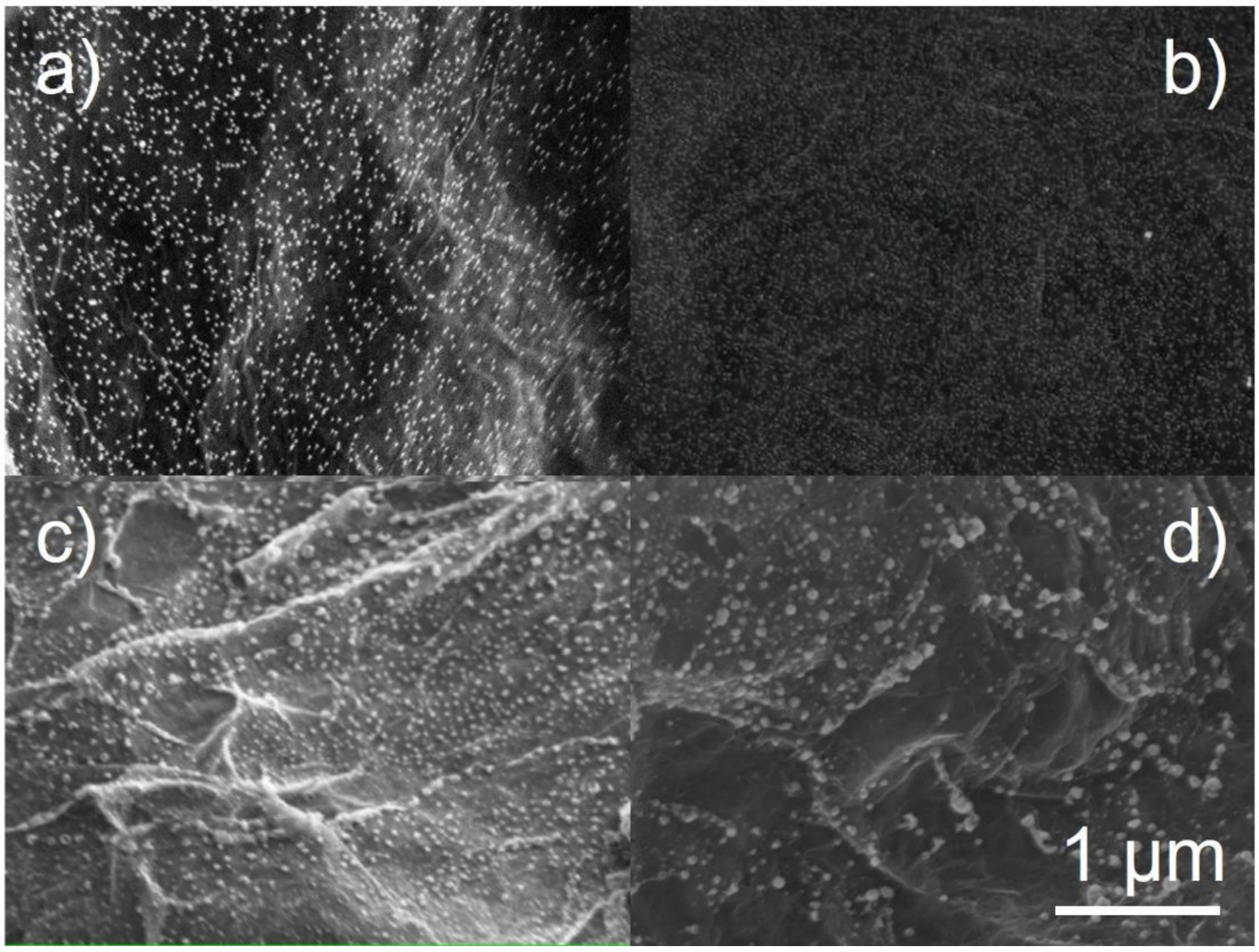

Fig. S8. High-resolution Au4f XPS spectrum of the Au@rGO catalyst acquired after the photothermocatalytic CO_2 conversion tests.

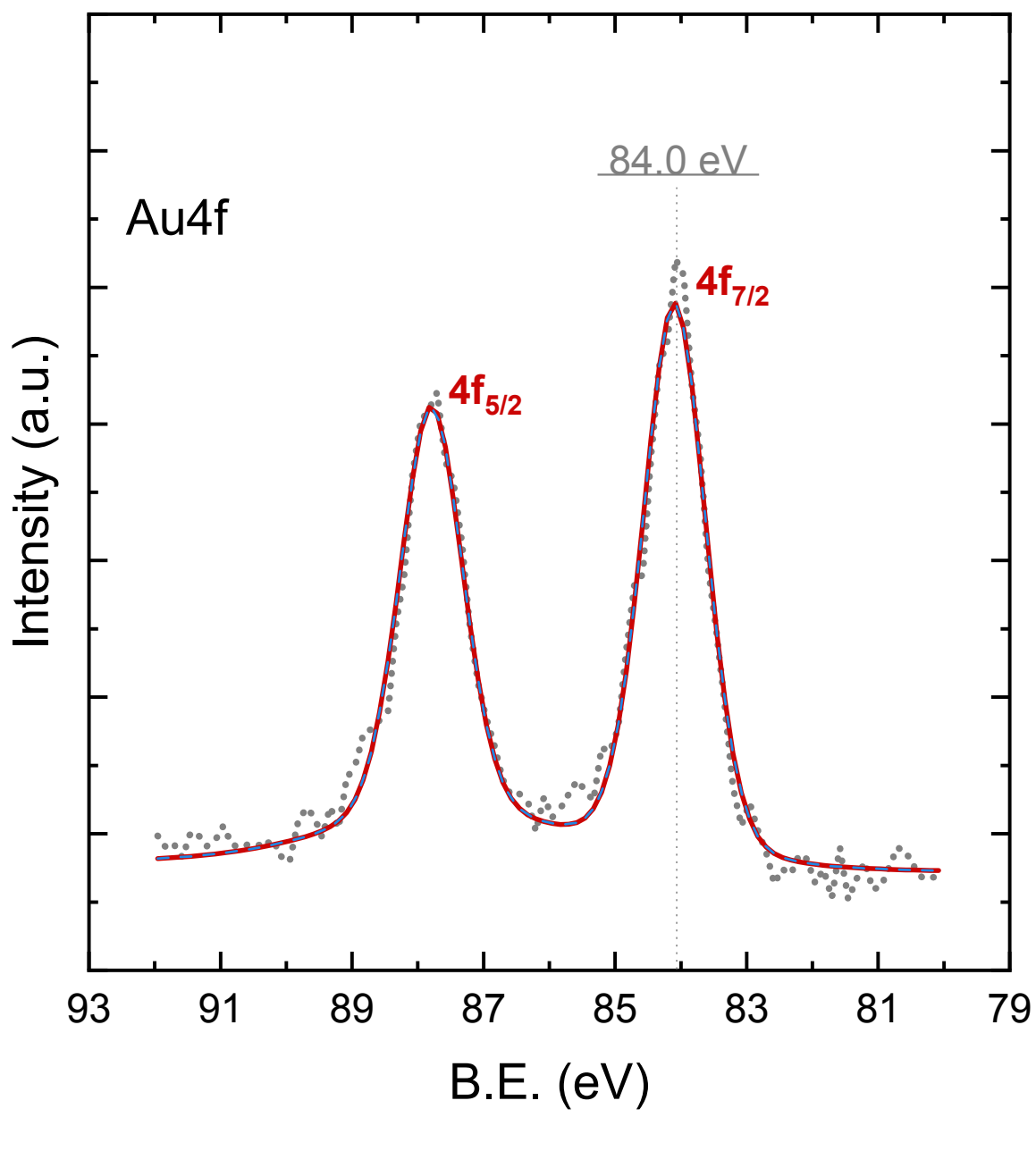

Fig. S9. High-resolution Cu2p XPS spectrum of the Cu@rGO catalyst acquired after the photothermocatalytic CO_2 conversion tests.

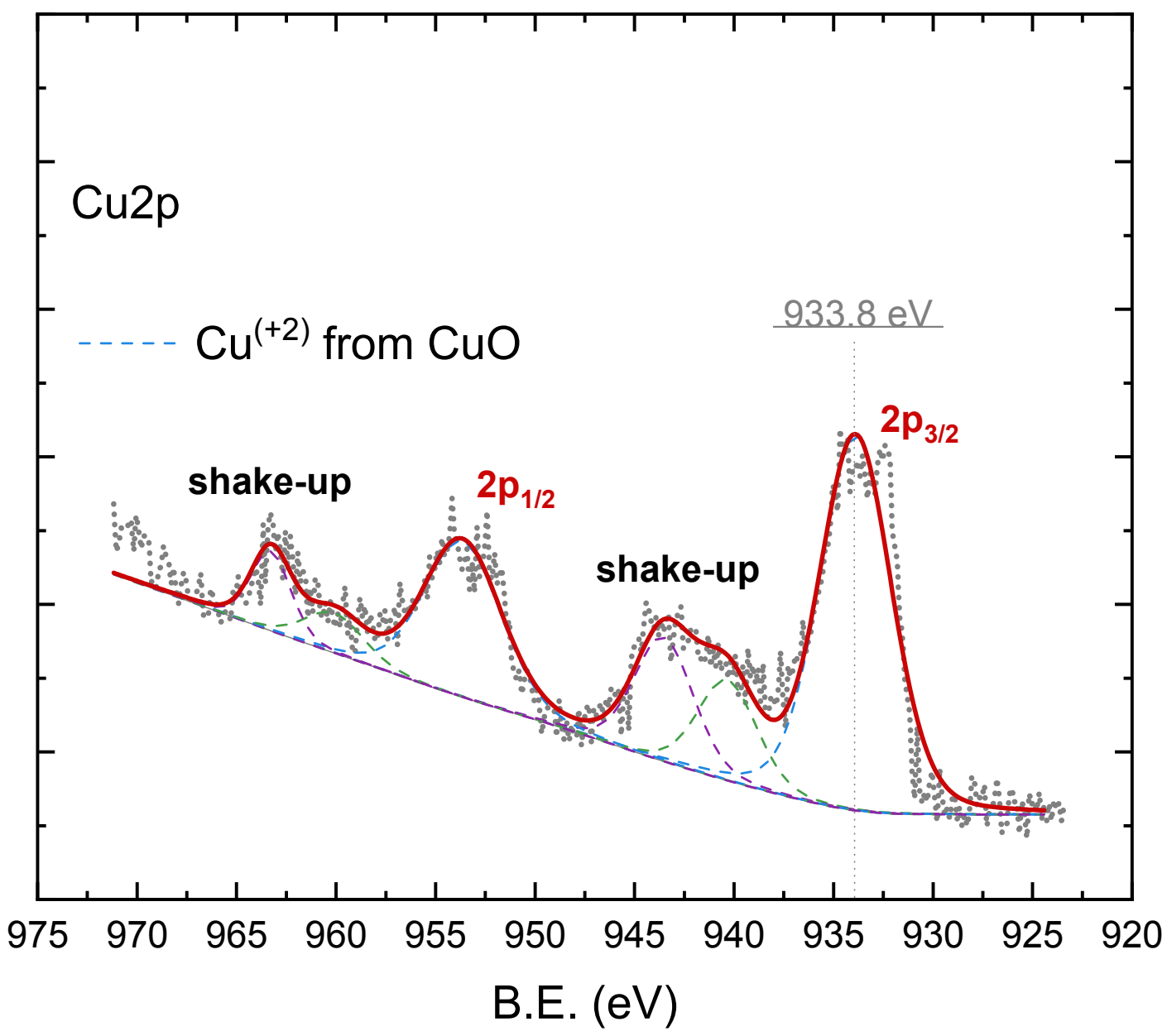

Supplement: Supplementary file 1 [file mg5c00221_si_001.pdf]
